# Retinal Ischemia as a Presenting Ocular Sign of Neurofibromatosis Type 2

**DOI:** 10.1155/2024/9133929

**Published:** 2024-01-23

**Authors:** Binbin Zhao, Yan Yan

**Affiliations:** Department of Ophthalmology, Renji Hospital, Shanghai Jiao Tong University, School of Medicine, Shanghai, China 200127

## Abstract

*Purpose.* Specific retinal abnormalities of neurofibromatosis type 2 (NF2) commonly include retinal astrocytoma and combined hamartoma of the retina and retinal pigment epithelium. Vasculopathy is an uncommon manifestation of NF2. We reported an NF2 patient presenting with retinal ischemia. *Observations.* An 18-year-old healthy Chinese female with acute decreased vision. The fundus examination and optical coherence tomography revealed optic disc hamartoma in the right eye and paracentral acute middle maculopathy (PAMM) and cotton wool spot indicating retinal ischemia in the left eye. Brain MRI showed bilateral acoustic neuroma, parasellar meningioma, and cervical extramedullary tumor. The genetic test confirmed the diagnosis of NF2. *Conclusions and Importance.* Our case suggests that retinal ischemia could be the presenting sign of NF2. NF2 could be associated with retinal vasculopathy in addition to retina tumors.

## 1. Introduction

Neurofibromatosis type 2 (NF2) is an autosomal-dominant multiple neoplasia syndrome that results from mutations in the NF2 tumor suppressor gene (Merlin) on chromosome 22q12. Patients are predisposed to the development of nervous system tumors, peripheral neuropathy, ophthalmological lesions, and cutaneous tumors [1]. Specific retinal abnormalities, including retinal astrocytoma and combined hamartoma of the retina and retinal pigment epithelium (CHRRPE), are commonly associated with NF2 [2]. Vasculopathy is an uncommon manifestation of NF2. Here, we reported a rare case of NF2 presenting as retinal ischemia in a Chinese adolescent.

## 2. Case Presentation

**Figure 1:**
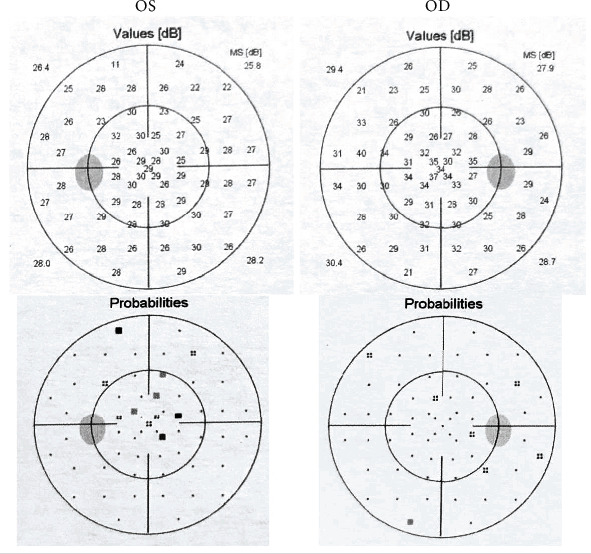
Visual field test for a young female patient with neurofibromatosis type 2 demonstrated a small central scotoma in the left eye.

**Figure 2:**
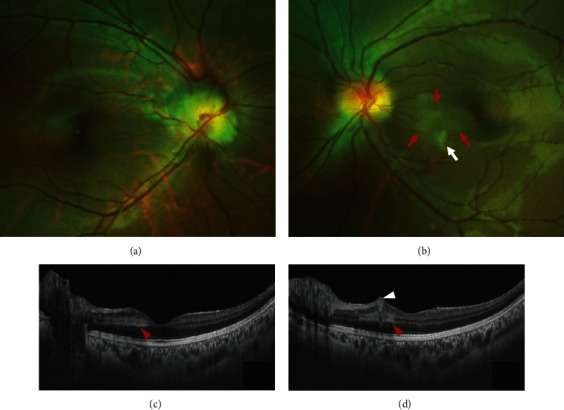
Fundus imaging and optical coherence tomography (OCT). (a) Swollen optic disc in the right eye. (b) Cotton wool spot (white arrow) and grey-white retinal lesion (red arrows) in the left eye. (c) OCT of the left eye shows the hyperreflective bands in the inner nuclear layer (red arrowhead). (d) OCT of the left eye shows the hyperreflective lesion (white arrowhead) in the superficial layer of the retina and hyperreflective bands in the inner nuclear layer (red arrowhead).

**Figure 3:**
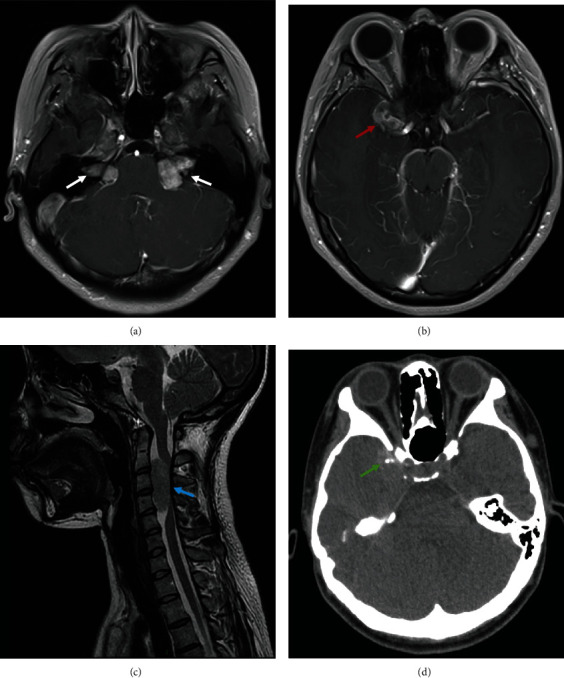
Neuroimaging of the patient with neurofibromatosis type 2. (a) Bilateral solid masses in the cerebellopontine angles on axial enhanced MRI (white arrows). (b) Axial enhanced MRI shows a parasellar lesion (red arrow). (c) Sagittal T2-weighted MRI of the spine shows a focal hypointense abnormality at the C2-C4 level (blue arrow). (d) Brain CT shows calcified lesions in the right temporal lobe (green arrow) and normal optic nerves.

An 18-year-old Chinese female noticed a grey dot in the central vision of the left eye four days ago. She had skin plexiform neurofibroma in the arm confirmed by a biopsy at another hospital a year before presentation. She denied any other previous medical history, trauma, and family history. She did not drink coffee or tea or smoke. She did not take any illicit drugs or oral contraceptive pills. On examination, her blood pressure was 110/70 mmHg. Her visual acuity was 20/20 in the right eye and 20/25 in the left without a relative afferent pupillary defect. Slit-lamp examination showed mild subcapsular cataracts in both eyes. Automated perimetry (Haag-Streit International, Koeniz, Switzerland; G1 TOP test strategy) revealed a small central scotoma in the left eye (Figure 1). Fundus examination revealed a swollen optic disc in the right eye, parafoveal grey-white deep retinal lesion, and cotton wool spot (CWS) in the posterior pole of the left eye (Figures 2(a) and 2(b)). FA revealed a focal filling defect of the retina corresponding to the CWS and hypofluorescence and no leakage of the right optic disc, which demonstrated retinal ischemia in the left eye and optic disc hamartoma in the right eye. Swept-source optical coherence tomography (SS-OCT) of the macula of the left eye demonstrated the hyperreflective bands in the inner nuclear layer consistent with paracentral acute middle maculopathy (PAMM) (Figures 2(c) and 2(d)). The carotid artery Doppler ultrasound and cardiac ultrasound were normal. The other laboratory investigations were unremarkable, including complete blood count with differential and platelet, liver enzymes, serum creatinine, lipid profile, erythrocyte sedimentation rate, C-reactive protein, blood glucose, hemoglobin A1C, antinuclear antibody, antidouble-stranded DNA antibody, antineutrophil cytoplasm antibody, syphilis, HIV, tuberculosis, toxoplasmosis, and angiotensin-converting enzyme. Thrombophilia (including deficiency of protein C, protein S, and antithrombin III) and homocystinuria were also excluded. The brain magnetic resonance angiography was unremarkable. The brain MRI with and without enhancement showed bilateral acoustic neuroma, parasellar meningioma, and cervical extramedullary tumor (Figures 3(a)–3(c)). Because she had an allergic reaction during the process of the brain MRI, the enhanced orbital MRI was deferred. Orbital CT revealed normal contours of bilateral optic nerves and calcified lesions in the right temporal lobe (Figure 3(d)). The genetic test revealed a heterozygous mutation (c.970delC (p.Q324Rfs^∗^22)) in the NF2 gene consistent with the diagnosis of NF2. Other family members did not agree to the genetic testing. The hearing testing and vestibular function were normal. The patient reported improved scotoma and visual acuity after a 3-month follow-up, and focal thinning of the retina on OCT in the left eye was noted.

## 3. Discussion

Our patient presented with a central scotoma due to both superficial and mid-retinal capillary ischemia on a background of NF2. NF2 is a familial tumor predisposition syndrome characterized by the development of bilateral vestibular schwannomas. Younger patients present predominantly nonvestibular symptoms. Several optic nerve and retinal manifestations are described in NF2, such as lens opacities, retinal tufts, epiretinal membranes, retinal hamartomas, and optic disc glioma.

Retinal vascular abnormalities are relatively common manifestations in NF1 [3], ranging from vascular tortuosity of arteries and veins to cork-crew patterned veins [4]. There are case reports of ocular ischemia associated with moyamoya syndrome in NF1 [5], but this situation was excluded from MRA in our patient. Unlike NF1, retinal vasculopathy is not typically associated with NF2. Only one case report revealed cilioretinal artery occlusion associated with papilledema in a patient with NF2. They proposed that the marked optic disc swelling elevated the pressure in cilioretinal circulation [6].

Although, to the best of our knowledge, retinal microvascular compromise associated with NF2 had not been reported before, brain stem ischemia and cerebral aneurysm have only rarely been described. Gugel et al. identified five young NF2 patients with aneurysms or ischemic stroke, and most of them occurred as the first symptom of the disease [7]. Lascelles et al. also reported three cases of ischemic stroke in children with NF2. They speculated that merlin, as a tumor suppressor protein, plays a role in vascular endothelial development so that the disease may break the vascular homeostasis between angiogenesis and antiangiogenesis [8].

CWS and PAMM represent an underlying process of inner and mid-retinal ischemia [9]. They are associated with retinal vascular compromise. Our patient was a young, healthy female, and she did not report any drug abuse or medication use which could be related to retinal ischemia. We thoroughly checked this patient and failed to find any vasculopathy risk factors except NF2.

In conclusion, our case suggests that retinal ischemia could be the presenting sign of NF2. NF2 could be associated with retinal vasculopathy in addition to retina tumors.

## Data Availability

The data used to support the findings of this study are available from the corresponding author upon request.

## Abbreviations

CHRRPE: Combined hamartoma of retina and retinal pigment epithelium
CWS: Cotton wool spot
NF: Neurofibromatosis
OCT: Optical coherence tomography
PAMM: Paracentral acute middle maculopathy.
